# The Circadian Molecular Machinery in CNS Cells: A Fine Tuner of Neuronal and Glial Activity With Space/Time Resolution

**DOI:** 10.3389/fnmol.2022.937174

**Published:** 2022-07-01

**Authors:** Francesca Fagiani, Eva Baronchelli, Anna Pittaluga, Edoardo Pedrini, Chiara Scacchi, Stefano Govoni, Cristina Lanni

**Affiliations:** ^1^Institute of Experimental Neurology, IRCCS San Raffaele Hospital and Vita-Salute San Raffaele University, Milan, Italy; ^2^Department of Drug Sciences, Pharmacology Section, University of Pavia, Pavia, Italy; ^3^Department of Pharmacy (DiFar), School of Medical and Pharmaceutical Sciences, University of Genoa, Genoa, Italy; ^4^Center of Excellence for Biomedical Research, 3Rs Center, University of Genoa, Genoa, Italy; ^5^Centro 3R (Inter-University Center for the Promotion of the 3Rs Principles in Teaching and Research), Italy

**Keywords:** circadian rhythms, microglia, astrocyte, neurotransmitters, brain areas, neuron

## Abstract

The circadian molecular machinery is a fine timekeeper with the capacity to harmonize physiological and behavioral processes with the external environment. This tight-knit regulation is coordinated by multiple cellular clocks across the body. In this review, we focus our attention on the molecular mechanisms regulated by the clock in different brain areas and within different cells of the central nervous system. Further, we discuss evidence regarding the role of circadian rhythms in the regulation of neuronal activity and neurotransmitter systems. Not only neurons, but also astrocytes and microglia actively participate in the maintenance of timekeeping within the brain, and the diffusion of circadian information among these cells is fine-tuned by neurotransmitters (e.g., dopamine, serotonin, and γ-aminobutyric acid), thus impacting on the core clock machinery. The bidirectional interplay between neurotransmitters and the circadian clockwork is fundamental in maintaining accuracy and precision in daily timekeeping throughout different brain areas. Deepening the knowledge of these correlations allows us to define the basis of drug interventions to restore circadian rhythms, as well as to predict the onset of drug treatment/side effects that might promote daily desynchronization. Furthermore, it may lead to a deeper understanding of the potential impacts of modulations in rhythmic activities on the pace of aging and provide an insight in to the pathogenesis of psychiatric diseases and neurodegenerative disorders.

## Introduction

**Figure 1:**
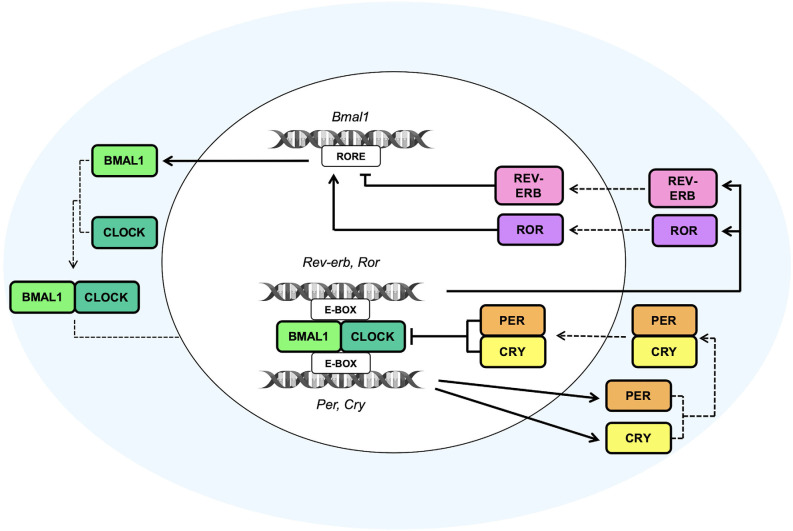
The molecular clock machinery. Schematic view of the molecular transcriptional–translational feedback loops (TTFLs) that coordinate the multiple cellular clocks across the mammalian body. The heterodimeric complexes given by the brain and muscle Arnt-like protein 1 (*Bmal1*) and circadian locomotor output cycles kaput (*Clock*) bind the Enhancer Box response element (E-box) on Period (*Per*) and Cryptochrome (*Cry*) gene promoters to induce the daytime expression of Per and Cry proteins, which, in turn, inhibit the transcriptional activity of Clock:Bmal1 heterodimer. The subsequent nocturnal degradation of Per and Cry proteins suppresses their inhibitory effect on Clock:Bmal1 heterodimer and allows to start a new oscillatory cycle. An additional feedback loop, consisting of the nuclear receptors *Rev-erb* and the retinoic acid orphan receptors *Ror*, is involved in the establishment of the rhythmic expression of *Bmal1* throughout the day. *Ror* and *Rev-erb* are both transcribed by the binding of the heterodimer Clock and Bmal1 to the E-box sequences on their gene promoters. Particularly, *Ror* positively regulates the transcription of the *Bmal1* gene, whereas *Rev-erb* acts as a transcriptional repressor of its expression, by both competing for binding Retinoic acid receptor-related Orphan Receptor Element (RORE) sites present on the *Bmal1* gene promoter. The cooperation and synchronization of all of these loops, that are the basis of the molecular clock machinery, contribute to the generation of daily rhythm.

Circadian rhythms are the basis of daily life, enabling the adaptation and adjustment of physiological and behavioral processes—from the brain to the peripheral organs—with the external environment. The entrainment and synchronization of the endogenous circadian oscillator rely on external timing cues, also called *Zeitgebers*, which allow the internal molecular clockwork to align with the environment. Light represents the main external cue that, through the retinohypothalamic tract (RHT; Gooley et al., 2001; Chen et al., 2011), is transmitted to the hypothalamic suprachiasmatic nucleus (SCN), the master circadian pacemaker (Stephan and Zucker, 1972; Ralph et al., 1990; Hastings et al., 2003). The SCN contains approximately 10,000 neurons, each of them displaying a cell-autonomous circadian clock that is able to coordinate all the other “slave” and peripheral oscillators located in nearly every mammalian organ. Noteworthy, the synchronization of peripheral clocks is not only hierarchically orchestrated by the hypothalamic circadian pacemaker through direct neuronal innervations but also through multiple soluble molecular signals, *i.e*., daily rhythms of glucocorticoids (Dibner et al., 2010). At the molecular level, the internal cellular clockworks are known as autoregulatory transcriptional–translational feedback loops (TTFLs; as schematized in Figure 1). The circadian locomotor output cycles kaput (*Clock*) and the brain and muscle Arnt-like protein 1 (*Bmal1*) are central players in the regulation of this loop. As positive regulators of the circadian cycle, they interact together to form the Clock:Bmal1 heterodimer in the cytoplasm. The latter translocates into the nucleus where it binds the Enhancer Box (E-box) response elements on the promoters of *Period* (*Per1, Per2, Per3*) and *Cryptochrome* (*Cry1, Cry2*), the core clock components of the negative arm of the TTFL (Hastings et al., 2018; Cox and Takahashi, 2019). Upon translation and nuclear accumulation, Per and Cry proteins inhibit the transcriptional activity of Clock:Bmal1 heterodimer. During night-time hours, Per and Cry proteins are progressively phosphorylated by the casein kinase 1ε/δ (CK1ε/δ) and adenosine 3’,5’-monophosphate (AMP) kinase (AMPK) respectively, ubiquitinated by their specific E3 ubiquitin ligase complexes, and finally degraded by the proteasome (Shirogane et al., 2005), thus turning off their inhibitory effect on Clock:Bmal1 and allowing for the onset of a new oscillatory cycle (Koike et al., 2012). In addition to the core loop, further regulators are represented by the nuclear receptors *Rev-erbα* (also known as *Nr1d1*), *Rev-erbβ* (also known as *Nr1d2*), and the retinoic acid orphan receptors *Rorα*, *Rorβ*, and *Rorγ* (*Rorα/β/γ*), which together form a second TTFL that steadies the robustness of the main TTFL, by regulating the rhythmic oscillation of *Bmal1* expression throughout the day. In particular, *Rorα/β/γ* positively regulate the transcription of *Bmal1* gene, whereas *Rev-erbα/β* act as transcriptional repressors of its expression, by both competing for binding to Retinoic acid receptor-related Orphan Receptor Element (RORE) sites present on *Bmal1* gene promoter (Preitner et al., 2002; Koike et al., 2012).

At the same time, also the intracellular calcium influx, membrane depolarization, and cAMP signaling, which all follow a daily rhythmical pattern, play a key role in the regulation of the biological transcriptional clock and in the establishment of the neuronal firing rhythms (Lundkvist et al., 2005; O’Neill et al., 2008). Indeed, through a cascade pathway these additional players are able to induce the phosphorylation of the calcium/cAMP response element binding protein (CREB) which, in turn, binds calcium/cAMP regulatory elements (CREs) sequences present on *Per1* and *Per2* gene promoters (Travnickova-Bendova et al., 2002; O’Neill et al., 2008), thus regulating their transcription and creating additional positive feedback loops involved in the generation of daily rhythm.

The pathways described above, however, are not the only mechanisms involved in the generation of rhythm. The TTFL, in fact, regulates the oscillations of approximately 20% of all the genome (Panda et al., 2002). Many other levels of regulation, such as post-transcriptional, translational, and post-translational modifications, play a critical role in coordinating rhythmicity. These modifications impact cellular localization, protein stability, and protein-protein interactions, and are essential for the modulation of multiple biological responses and for the establishment of a fine-tuning rhythmicity (Brenna and Albrecht, 2020). Besides, alterations or mutations of the kinases or on the target sequences responsible for these post-translation modifications can result in several diseases strictly linked to the circadian system, such as sleep disorders, neurodegenerative, and psychiatric diseases, and also cancer (Schwab et al., 2000; Xu et al., 2005; Fang et al., 2015; Brenna and Albrecht, 2020).

Since the circadian molecular machinery acts as a temporal variable, orchestrating a number of physiological functions and processes in the central nervous system (CNS), it is fundamental to acquire an understanding of the molecular mechanisms regulated by the clock in the different brain areas and within the different CNS cells. Therefore, in this review, we aim to critically discuss evidence regarding the role of circadian rhythms in the regulation of neuronal activity and neurotransmitter systems.

## The Circadian Electrical Activity

In the SCN, circadian timekeeping is a dynamic process characterized by reciprocal interactions between the molecular oscillations of the clock machinery and the electrical activity of neurons. TTFLs are able to orchestrate, in a circadian manner, the variations of Na^+^ and K^+^ currents (Flourakis et al., 2015), as well as the intracellular concentration of Ca^2+^ ([Ca^2+^]_i_; Brancaccio et al., 2013), responsible, in turn, for the regulation of neuronal activity. Remarkably, the [Ca^2+^]_i_, moving into cells through voltage-operated calcium channels activated by electrical firing (VOOC), NMDA-type glutamate receptors (NMDARs), or released by intracellular stores (Harvey et al., 2020), plays a pivotal role in the modulation of the biological clockwork (Brancaccio et al., 2013). Indeed, the electrical activation due to the peak of [Ca^2+^]_i_ triggered by a light signal has been found to induce the expression of *Per1* and *Per2* genes, thereby affecting the expression of the positive TTFL components (Brancaccio et al., 2013). Notably, at circadian time 6 (CT6) light signals induce Ca^2+^ influx and activate a phosphorylation cascade pathway involving the protein kinase A and G (PKA and PKG), the Ca^2+^/calmodulin-dependent protein kinase (CaMK), the mitogen-activated protein kinases (MAPK) and the extracellular-signal-regulated kinases (ERK). This pathway leads to the phosphorylation of CREB, which, after being activated by the cAMP-regulated transcriptional co-activator 1 (CRTC1), heterodimerizes with the histone acetyltransferase CREB binding protein (CBP) and then, at CT9, binds the CRE sequences present on *Per* gene promoters (Gau et al., 2002; Parra-Damas et al., 2017), leading to the consequent transcription of *Per1 (*at CT10) and *Per2* (at CT12; Brancaccio et al., 2013). Moreover, Brenna et al. recently demonstrated that Per2 supports the stimulus-dependent induction of the *Per1* gene by regulating the CREB/CRTC1/CBP complex. Accordingly, upon light or forskolin stimuli, Per2 acts as a co-factor of CREB promoting the formation of a transactivation complex on the CRE element of the *Per1* gene. In particular, Per2 has been shown to facilitate the interaction between CREB and its co-regulator CRTC1, thereby inducing complex formation (Brenna et al., 2021).

**Figure 2:**
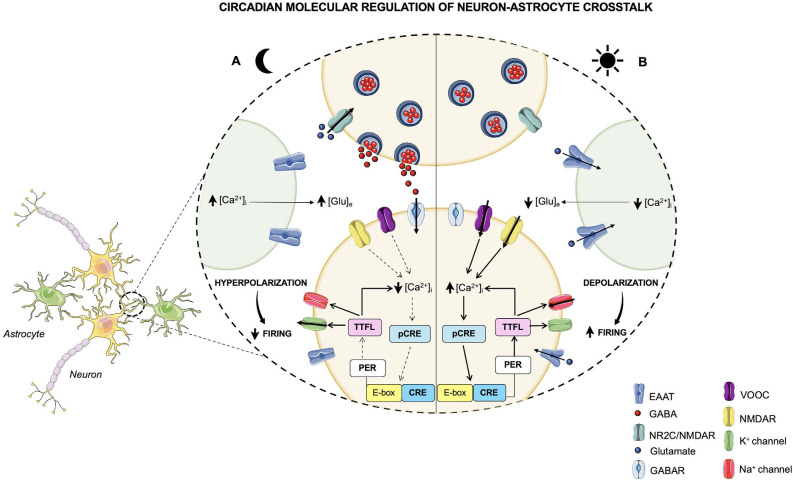
Circadian molecular regulation of neuron-astrocyte crosstalk. The circadian timekeeper in the SCN is dictated by reciprocal interactions between the molecular oscillations of the clock machinery and the electrical activity of neurons, which lead to the establishment of a daily rhythm. **(A)** During the night, astrocytes are active and their [Ca^2+^]_i_ is high, leading to an increased release of extracellular Glu, which, in turn, through the NR2C/NMDA receptors, is able to induce the release of GABA from presynaptic neurons. GABA acts on the postsynaptic GABA receptors and exerts its inhibitory effect on neurons that during the night are less active. In fact, the neuronal [Ca^2+^]_i_ is low, and, as a result, the loop that impacts the TTFLs is blunted. However, the TTFL activates the K^+^conductances, thus leading to a hyperpolarization of the cell membrane and to a dampened firing. **(B)** During the daytime, when astrocytes are quiescent with a low [Ca^2+^]_i_, Glu is re-uptaken by astrocytes and neurons and, consequently, its extracellular levels are decreased. On the other hand, neurons are active and neuronal [Ca^2+^]_i_, whose oscillations are controlled by the TTFL, is high. The neuronal electrical activity stimulates the activation of the Ca^2+^/cAMP-responsive elements (CREs) on *Per* genes, thus promoting the transcription of *Per1* and *Per2* genes. Subsequently, the transcription of these TTFL components leads to a further rise in the [Ca^2+^]_i_ and to the activation of Na^+^ currents that results in cell membrane depolarization and a heightened firing.

Furthermore, since the release of [Ca^2+^]_i_ follows a circadian pattern orchestrated by the TTFLs, the bidirectional interlink between the clockwork and the neural signaling creates a closed loop that drives the rest-activity cycle of the neuronal activity (Brancaccio et al., 2013; Figure 2). These findings demonstrate that TTFL is strongly sensitive to the electrical state, and that the coupling between the clock machinery and Ca^2+^ activity is fundamental for a proper generation of rhythm and for a robust timekeeping within the brain. Hence, the electrical activity seems to play a key role in re-setting the intracellular clock, contributing to the suppression of neuronal activity during the night, and to the induction of a subsequent electrical firing peak with the coming of the next circadian day.

## The Clock in Neurotransmission

Data concerning the synthesis and the uptake of neurotransmitters and their correlation with the expression/functions of circadian pacemakers support the role of certain transmitters (*e.g*., dopamine -DA-, serotonin -5-HT-, and γ-aminobutyric acid -GABA-) as fine-tuners of daily rhythmicity (Terazono et al., 2003; Imbesi et al., 2009; Nakamaru-Ogiso et al., 2012; Barca-Mayo et al., 2017; Brancaccio et al., 2017; Maejima et al., 2021; Palm et al., 2021). Deepening the knowledge of these correlations may lay the foundation of drug interventions aimed at restoring circadian rhythms, but also at predicting the onset of side effects related to drug treatment/abuse that might cause daily desynchronization. Besides the events discussed so far, other specific mechanisms dictating the strength and the specificity of chemical transmission would deserve attention due to their potential role in circadian rhythmicity. In relation to the latter, we will briefly discuss how the mode of synaptic communication, the co-localization of different neurotransmitters, and the intensity of the releasing stimulus (Albers et al., 2017) may be relevant to the rhythmicity of the neurotransmitter-clock protein crosstalk.

Regarding the synaptic communication, catecholaminergic and indolaminergic projections originate from a limited number of neurons in defined central areas, *i.e*., the *locus coeruleus* (LC) for noradrenaline (NA), the *substantia nigra* (SN), and the ventral tegmental area (VTA) for DA or the *raphe nucleus* for 5-HT (Descarries and Mechawar, 2000; Vizi et al., 2004). Once released, the neurotransmitter diffuses through the biophase to reach receptors located postsynaptically, a process named “volume transmission” to assure the communication among sites within the same brain area or even in adjacent brain regions (Fuxe et al., 2012; Olivero et al., 2020). In this regard, DA, which controls the daily rhythmicity in mammals (Imbesi et al., 2009; Hood et al., 2010), is actively released from dopaminergic projections and reaches the postsynaptic structures by “volume transmission” (Descarries and Mechawar, 2000), which are located also in vicinal regions (*i.e*., the striatum, the SCN). Here, the activation of dopaminergic receptors (*i.e*., the D1 and D2 receptors) elicits functional responses, including the tuning of clock proteins, *i.e*., the Per1 and 2 proteins (Imbesi et al., 2009; Hood et al., 2010). Therefore, DA diffusion allows the propagation of the dopaminergic input during the diurnal period, favoring a widespread circadian regulation of biological processes. However, the efficiency of the DA “volume transmission” strictly depends on the continuous replenishment of the presynaptic vesicle, that is ensured by the storage of the newly-synthetized transmitter or by the re-uptake of the released amine, which are both efficiently controlled by clock proteins (see Section “The Dopaminergic System”). Besides the SN and the VTA, dopaminergic cells also represent the largest percentage of the resident cells in the retina. Here, the highest concentration of DA is reached during the day due to multiple excitatory signals, including light, and it is controlled by melatonin. DA diffuses by “volume transmission” also in the brain regions to enable the communication between the amacrine cells (that are dopaminergic in nature) and the horizontal and the bipolar cells, as well as the photoreceptor-containing processes (Witkovsky, 2004). The mechanism of “volume transmission” in the CNS dictates a virtuous “clock protein-transmitter loop” that favors the diurnal control of some circadian regulators. An interesting feature of the retinal amacrine cells (Hirasawa et al., 2009) and in general of the DA-positive neuronal processes in the SCN and striatum (Romei et al., 2016), is that these cells are also specialized for GABA. Dopaminergic cells cannot synthesize GABA, but can efficiently recapture this amino acid, mainly at nerve endings (Romei et al., 2016), where it is co-stored with DA. The uptake of GABA in dopaminergic terminals, which is an event that would occur preferentially when the [GABA]_out_ exceeds the physiological level, triggers a concomitant release of the catecholamine. Since GABA acts preferentially as a nocturnal controller of circadian rhythms and has an opposite outcome with respect to DA in controlling the expression of the clock genes [*i.e*., *Per2* (Ruan et al., 2008)], it is possible that the GABA-induced carrier-mediated release of DA may have a role in permitting the transition from the nocturnal to the diurnal phase of the daily circadian rhythmicity.

The SCN is composed of GABAergic neuronal subtypes and glial cells. As well as many neurons, a large part of the GABAergic SNC neurons also contains neuropeptides. Therefore, depending on the stimulus, these neurons preferentially release both GABA and neuropeptides. When the concomitant release of neuropeptides and GABA occurs, this may affect the GABA outcomes, causing an indirect impact on the daily timekeeping due to the modifications of the hierarchical organization of the neuronal control of the clock oscillation. Interestingly, the co-release of neuropeptides and GABA in the SCN and in some other hypothalamic regions has been recently related to the dyssynchronization of the clock circadian molecules, particularly *Clock* and *Period*, that occurs in stress conditions (Wong and Schumann, 2012).

In the mammalian brain, serotonin availability in the biophase varies following a circadian pattern from day to night. The serotonergic projections from the medial *raphe nucleus* converge in the SCN and in the retino-hypothalamic tract, where the indolaminergic signal is mainly mediated by presynaptic 5-HT1B receptors (Selim et al., 1993; Pickard et al., 1999; Smith et al., 2001). 5-HT1B receptors are also largely expressed in GABA terminals, particularly in the SCN neurons and in the RHT, where their activation inhibits the exocytosis of the inhibitory amino acid. 5HT modulates the effect of light on the clock at both early and late night (Morin, 1999) time points, with variable effects when applied at night. Furthermore, a recent study using a rat *in vivo* microdialysis revealed that diurnal changes in 5-hydroxyindole-acetic acid (5-HIAA) levels, the principal 5-HT metabolite, could be observed also in other brain regions that receive the serotonergic projections, in particular, in the prefrontal cortex (PFC), but not in the hippocampus; this metabolic activation might be related to enhanced neuronal activity of 5-HT (Daszuta and Barrit, 1982; Nakayama, 2002). The contribution of 5-HT to the expression and function of clock proteins might depend on the phase of the day and on brain areas but again largely involves the control of the GABAergic innervation.

Notably, based on evidence from the literature suggesting a functional crosstalk between neurotransmitter systems and the circadian clockwork, we will summarize evidence showing such bidirectional interplay (as schematized in Table 1). Accordingly, robustness and precision in daily timekeeping have been reported to rely on the punctual intercellular communication, mediated by neurotransmitters, among CNS cells (Herzog et al., 2004; Hastings et al., 2018).

**Table 1:** The clock around neurotransmission.

**Clock genes**	**Experimental model**	**Neurotransmitter players**	**Circadian interactions**	**References**
**RETINA**				
*Per1*	*mPer2^Luc^* mouse retinal explants	Retinal D1 receptors	Stimulation of D1 receptors results in the upregulation of the *Per1* gene *via* the activation of ERK-MAPK-CREB pathway.	Ruan et al. (2008)
*Per1*	*Mouse NG108-15 cells*	Retinal D2 receptors	Activation of D2 receptors leads to an increase in the transcription of the *Per1* gene *via* the activation of ERK-MAPK-CREB cascade.	Yujnovsky et al. (2006)
*Per2*	Cultured *mPer2*^Luc^ retinal explants	GABA-A and GABA-C receptors	The treatment with a GABA-A or GABA-C receptor agonist significantly suppresses *Per2* levels, probably acting on a posttranscriptional level, and partly by stimulating case in kinase.	Ruan et al. (2008)
*Per1, Bmal1*	Cultured *mPer2*^Luc^ retinal explants	GABAergic system	GABA co-administration is able to increase the mRNA levels of *Per1* and *Bmal1*.	Ruan et al. (2008)
**CORTEX**				
*Per1*	Mice	Noradrenergic system	Administration of NA highly induces *Per1* mRNA expression in the mice cerebral cortex during the light phase, probably through the activation of cAMP-PKA and MAPK-CREB pathways.	Burioka et al. (2008)
*Npas2, Clock*	Cortical astrocytes cultures from *Npas2* and *Clock* mutant mice	Glutamate transporter EAAT1	Mutation of *Npas2* and *Clock* results in a decrease in the mRNA levels of the glutamate transporter EAAT1.	Beaulé et al. (2009)
*Bmal1, Cry1, Per2*	Mouse cortical neurons	GABA-A receptors	The activation of GABA-A receptors is able to entrain rhythmic oscillations of *Bmal1*, *Cry1*, and *Per2* expression that, in turn, is completely suppressed inhibiting them.	Barca-Mayo et al. (2017)
*Bmal1*	*Bmal1*cKO mice	GABA transporters Gat1 and Gat3	The KO of *Bmal1* in astrocytes significantly decreases the expression of Gat1 and Gat3 in the cortex.	Barca-Mayo et al. (2017)
**STRIATUM**				
*Per2*	*Per2*^Brdm1^ mutant mice	MAO-A gene	*Per2* appears to induce the transcription of MAO-A gene, probably due to its action on the E-boxes of the MAO-A transcriptional promoter.	Hampp et al. (2008)
*Per2*	Male Wistar rats	D2 receptors	D2 antagonists decrease the rhythmical amplitude of *Per2* transcription, that is, in turn, restored by D2 agonists, thus indicating a key role of D2 receptors in the regulation of *Per2* gene expression, probably *via* the activation of ERK-MAPK-cAMP-CREB cascade.	Hood et al. (2010)
*Per1, Bmal1, Clock, Npas2*	Cultured mouse striatal neurons	D1 receptors	Treatments with D1 receptor agonists lead to an increase in mRNA levels of *Per1, Bmal1, Clock, and Npas2*.	Imbesi et al. (2009)
*Per1, Clock*	Cultured mouse striatal neurons	D2 and D3 receptors	Treatments with D2 and D3 receptor agonists repress the transcription of *Per1* and *Clock* genes.	Imbesi et al. (2009)
*Rorα*	Mouse	D3 receptors	*Rorα* upregulates D3 receptor transcription probably due to its binding to the DRD3 promoter.	Ikeda et al. (2013)
*Rev-erbα*	Mouse	D3 receptors	*Rev-erbα* periodically inhibits D3 receptor transcription probably due to its binding to the DRD3 promoter.	Ikeda et al. (2013)
*Per2*	G*Per2* mutant mice	D3 receptors	Deletion of *Per2* in glial cells shows an increase in D3 receptor mRNA levels.	Martini et al. (2021)
*Per2*	G*Per2* mutant mice	GABA transporter	Gat2 Deletion of *Per2* in glial cells increases the expression of *Gat2* in the NAc.	Martini et al. (2021)
**VTA**				
*Rev-erbα*	*Rev-erbα* knockout (RKO) mice Immortalized DAergic cell lines (CATH.a)	TH gene	Suppressive mechanism of *Rev-erbα* on the regulation of TH gene expression, by acting on RRE/NBRE1 elements located in TH promoters.	Chung et al. (2014)
*Clock*	*Clock*Δ*19* mutant mice	TH gene	*Clock* suppresses the expression of TH gene, through a possible interaction of *Clock* with the E-boxes sequences on TH gene promoter.	McClung et al. (2005)
*Clock*	*Clock*Δ*19* mutant mice	Dopamine neurons	Deletion of the *Clock* leads to an increase in the bursting and firing rate of VTA dopamine neurons, probably induced by the down-regulation of a voltage-gated potassium channel (*KcnQ2*).	McClung et al. (2005)
**HYPOTHALAMUS**				
*Per2*	G*Per2* mutant mice	GABA transporter Gat1	Deletion of *Per2* in glial cells significantly decreases the expression of *Gat1* in the hypothalamus.	Martini et al. (2021)
**SCN**				
*Per2, Rev-erbα*	Sudanian grass rats	5-HT 5-HT receptors	Treatments with a 5-HT agonist and selective 5-HT reuptake inhibitor increase *Per2* and *Rev-erbα* mRNA levels, enhancing light-induced phase delays at CT12.	Cuesta et al. (2008)
*Per1, Rev-erbα, Rev-erbβ*	Sudanian grass rats	5-HT 5-HT receptors	Treatments with a 5-HT agonist and selective 5-HT reuptake inhibitor increase *Per1*, *Rev-erbβ*, and *Rev-erbα* mRNA levels, reinforcing light-induced phase-advances at CT0.	Cuesta et al. (2008)
*Rorβ*	Sudanian grass rats	5-HT 5-HT receptors	Treatments with a 5-HT agonist and selective 5-HT reuptake inhibitor decrease *Rorβ* transcription.	Cuesta et al. (2008)
*Per 2*	*Per2*^*Brdm1*^ mutant mice	Glutamate transporter EAAT1	Mutation of *Per2* leads to a reduction in the SCN expression of EAAT1, thus resulting in a hyper-glutamatergic state.	Spanagel et al. (2005)
*Per2*	Mouse SCN slices	Glutamate transporter EAAT3	Inhibition of EAAT3 transporter leads to an increase of the extracellular Glu levels, which consequently causes a dramatically reduction of the amplitude and robustness of *Per2* gene oscillations.	Brancaccio et al. (2017)
*Per2*	Mouse SCN slices	NR2C subunits of the NMDA receptors	NR2C inhibition abolishes circadian oscillations of intracellular Ca^2+^, provokes nighttime depolarization of SCN dorsal neurons, and reduces the amplitude and lengthens the period of *Per2* oscillations.	Brancaccio et al. (2017)
*Per2*	Avp-Vgat^−/−^ mice	Vesicular GABA transporter (Vgat)	The deletion of the vesicular GABA transporter (Vgat) in arginine vasopressin-producing (AVP) neurons, leads to a delay in the peak phase of *Per2* oscillations resulting in a lengthened activity time of mice circadian behavioral rhythms.	Maejima et al. (2021)
*Bmal1*	*Bmal1*cKO mice	GABA transporters Gat1 and Gat3	The deletion of astrocytic *Bmal1* leads to a noteworthy reduction of the expression of GABA transporters *Gat1* and *Gat3* in the SCN and in astrocytes.	Barca-Mayo et al. (2017)
**PERIPHERAL SYSTEM**				
*Per1*	Mice	Noradrenergic receptors	Activation of noradrenergic receptors by α/β-adrenoreceptor agonists increases m*Per1* expression in mice liver in a concentration-dependent manner.	Terazono et al. (2003)
*Per1, Per2, Bmal1*	SCN-lesioned arrhythmic mice	Noradrenergic system	Lesions of the SCN cause a loss of liver daily expression of m*Per1*, m*Per2*, and m*Bmal1* and of NA content.	Terazono et al. (2003)
*Bmal1, Per1, Per3*	Human dermal fibroblast	Noradrenergic system	The exposure to different NA concentrations was able to reduce the expression of *Bmal1* at ZT12 and *Per1* at ZT20, and to increase *Per3* mRNA levels at ZT4.	Palm et al. (2021)

### The Dopaminergic System

Dopamine (DA) is produced in midbrain neurons of the *substantia nigra* (SN) and ventral tegmental area (VTA) and it is involved in the regulation of locomotion, sleep-wake cycle, learning, motivation, and reward. DA levels have been shown to display a circadian oscillation in several brain areas, such as the retina, midbrain, striatum, olfactory bulb, and hypothalamus (Korshunov et al., 2017).

In particular, the retina is the first region of CNS to deal with light information and it is responsible for its transmission to SCN through the retino-hypothalamic pathway (Gooley et al., 2001; Hattar et al., 2006; Prigge et al., 2016). Studies on mammalian retinas show that DA has the highest peak of synthesis during the daytime, whereas it is reduced during the nighttime. Doyle et al. (2002) found that in rats with normal and dystrophic retinas, DA content and turnover, as observed by measuring its two major metabolites 3,4-dihydroxyphenylacetic acid (DOPAC) and homovanillic acid (HVA), follow a daily rhythmicity with the peak during the light phase. Besides, they also reported that cones and rodes are not required for generating DA circadian rhythmicity, since the latter was maintained also in rats with dystrophic retinas (Doyle et al., 2002). Moreover, dopamine has been reported to stimulate the expression of the core clock component *Per1* in retinal neurons, especially through the activation of D1 and D2 receptors, whose specific role is currently a matter of debate. In intact m*Per2*^Luc^ retinal explants, stimulation of D1 receptors resulted in *Per1* upregulation by activating the extracellular signal-regulated kinase (ERK)-mitogen-activated protein kinase (MAPK) pathway. Enhanced phosphorylation of the cAMP-responsive element-binding protein (CREB) has been found to promote the cAMP-response elements (CRE)-mediated transcription of *Per1* gene (Ruan et al., 2008). In line with such evidence, Yujnovsky et al. (2006) reported an increase in *Per1* transcription in mouse NG108–15 cells, induced by the activation of dopamine D2 receptor (D2R) by D2 agonists, such as quinpirole. Indeed, the stimulation of D2 receptors led to the activation of the MAPK transduction cascade, to an increase in CREB phosphorylation and the recruitment of the Clock:Bmal1 heterodimer, thus resulting in an enhanced *Per1* transcription. A marked reduction of the Clock:Bmal1 activation and, consequently, of *Per1* levels was also observed in the retina of D2R-null mice, thus indicating the importance of D2 receptors in mediating retinal circadian expression (Yujnovsky et al., 2006).

Besides the retina, the circadian activity of the dopaminergic signaling system has also been reported in the midbrain and striatum, possibly transmitted through the neuronal projections from the SCN to these areas, such as *via* the orexinergic system (ORX) in the lateral hypothalamus (LH), the *lateral habenula* (LHb), the paraventricular thalamic nucleus (PVT), and the medial preoptic nucleus (MLPO; Abrahamson and Moore, 2001; Mendoza and Challet, 2014). These innervations from the SCN are able to regulate DA firing, expression, and metabolism. Notably, the LHb acts as an inhibitory pathway on the VTA. Accordingly, its activation has been found to decrease the firing of dopaminergic neurons (Bourdy and Barrot, 2012; Lecca et al., 2012). Furthermore, activation of the ORX system in the VTA has been shown to be implicated in the regulation of the plasticity of the dopaminergic system, as well as in locomotion, reward, motivation, and addiction (Borgland et al., 2006; Narita et al., 2006). Consequently, the rhythmical dopaminergic activity in the striatum relies on this SCN-VTA pathway. All these findings suggest that the intricate circuit of interconnections between SCN and the different brain areas plays a pivotal role in establishing the proper circadian rhythmicity of the dopamine system in the VTA and in the striatum.

DA signaling, synthesis, transport, and degradation are all under circadian regulation. Promoters of genes encoding for tyrosine hydroxylase (TH, the rate-limiting enzyme of DA synthesis), dopamine transporter (DAT), and monoamine oxidase A (MAO-A, the key enzyme that regulates DA degradation) have been found to contain E-Box sequences and to display circadian rhythmicity in their expression (Yoon and Chikaraishi, 1994; Sleipness et al., 2007; Hampp et al., 2008). In this regard, Sleipness and coworkers (Sleipness et al., 2007) demonstrated that electrolytic lesions of the SCN in rats alter the day-night variations in the expression of the DA transporter and synthetase in the midbrain. Remarkably, many studies revealed that the firing of dopaminergic neurons and the expression of TH protein in the VTA change during the day, with a peak during the daytime (Webb et al., 2009). In particular, *Clock* and *Rev-Erbα* have been found to be implicated in the reduction of *TH* expression in the VTA (McClung et al., 2005; Chung et al., 2014). Notably, Chung et al. (2014) demonstrated, in *in vitro* and *in vivo* studies on wild-type and *Rev-erbα* knockout (RKO) mice, an increase in *TH* mRNA expression and DA extracellular content in RKO compared to WT mice. These findings indicate a loss of rhythmicity and a parallel hyper-activation of the dopaminergic system in RKO mice, suggesting that *Rev-erbα* may negatively regulate *TH* gene expression. In particular, *Rev-erbα* has been shown to control *TH* expression by acting on RRE/NBRE1 (NGFI-B response element) elements located in *TH* promoters through the recruitment of the histone deacetylase 3 (HDAC3) complexes. The latter negatively regulates permissive histone acetylation, resulting in the inhibition of the transcriptional activation of the *TH* gene promoter (Chung et al., 2014). Furthermore, McClung and coworkers revealed that *TH* expression in mice was downregulated by *Clock*. Indeed, *TH* mRNA expression and protein levels were strongly increased in the VTA of *Clock*Δ*19* mutant mice compared with WT (McClung et al., 2005). Although the mechanism by which *Clock* modulates *TH* expression is unknown, *Clock* has been suggested to bind E-box sequences on the *TH* gene promoter. Moreover, *Clock*Δ*19* mutant mice have shown an increase in bursting and firing rates of VTA dopamine neurons. Such an effect is probably mediated by the downregulation of a voltage-gated potassium channel (*KcnQ2*), induced by the loss of *Clock* in mutants, thus indicating the key implication of *Clock* in affecting the excitability of DA neurons (McClung et al., 2005).

In the striatum, the rhythmical DA and MAO-A content has been found to be regulated by *Per2*. *Per2*^Brdm1^ mutant mice have been observed to display a reduction in *MAO-A* expression in the striatum and a consequent increase in extracellular DA levels, thus suggesting that *Per2* may promote *MAO-A* transcription (Hampp et al., 2008). As a feedback loop, DA has been found to regulate the rhythm of *Per2* expression *via* activation of D2 receptors. Indeed, rats treated with selective D2 antagonists (*i.e*., raclopide) showed a significant decrease in the rhythmical amplitude of *Per2* transcription in the striatum, which, conversely, was not observed upon treatment with D1 antagonists. In turn, the reduction of *Per2* levels, induced by the administration of 6-hydroxydopamine (6-OHDA), was restored by using D2 agonists, such as quinpirole, whereas the use of D1 agonists had no effect (Hood et al., 2010).

Taken together, these results suggest that fluctuation in extracellular DA levels may be involved in the maintenance of the rhythmical amplitude of *Per2* transcription in the dorsal striatum *via* D2 receptors. Such modulation has been thought to involve the ERK-MAPK intracellular pathway, which is regulated by DA signaling, and leads to the activation of the cAMP-CREB pathway that results in the augmented transcription of *Per2* gene (Hood et al., 2010). Moreover, Imbesi and coworkers, investigating the implication of DA receptors in the regulation of neuronal clock genes expression in cultured mouse striatal neurons, found that treatment with D1 receptor agonists led to an increase in *Per1, Bmal1, Clock, and Npas2* mRNA levels. On the other hand, treatments with D2 and D3 receptor agonists, such as quinpirole, repressed the transcription of *Per1* and *Clock* genes, whereas *Npas2* and *Bmal1* mRNA expression was not affected (Imbesi et al., 2009). Additionally, in mouse ventral striatum, a 24-h variation in the expression of dopamine D3 receptor has been reported. Interestingly, *Rora* and *Rev-erbα* have been found to modulate these oscillations, by possibly binding the D3 receptor promoter. In particular, *Rora* has been found to upregulate D3 receptor transcription, whereas *Rev-erbα* has been reported to periodically inhibit its expression (Ikeda et al., 2013). Moreover, in the *nucleus accumbens* (NAc), D3 receptors have been found to be regulated also by glial *Per2* (Martini et al., 2021). Indeed, mice with a deletion of *Per2* in glial cells showed an increase in D3 receptor mRNA levels compared to control animals (Martini et al., 2021). In particular, *Per2* has been suggested to interact with several nuclear receptors, such as *Rev-erbα* and, to a lesser extent, with *Rora* (Schmutz et al., 2010), both interacting with the D3 receptor’s promoter and consequently modulating D3 receptor expression (Ikeda et al., 2013). Together, these studies highlight the putative links between the dopaminergic system and the striatal clockwork.

### The Serotonergic System

Serotonin (5-HT) is an important neurotransmitter involved in the coordination and synchronization of the circadian system and melatonin synthesis. The serotonergic system and the master pacemaker are interconnected with each other through several projections, which are probably at the root of their reciprocal regulation. SCN projects indirectly to the median *raphe nucleus* (MRN) and the dorsal *raphe nucleus* (DRN) *via* the dorsomedial hypothalamic nucleus (DMH). In turn, the *raphe nuclei* project directly back to the SCN from the MRN, whereas indirectly *via* the intergeniculate leaflet (IGL) from the DRN (Azmitia and Segal, 1978; Hay-Schmidt et al., 2003; Deurveilher and Semba, 2005).

Clock genes have been found to regulate the expression of genes implicated in the serotonergic pathway in a circadian manner. The *Tph2* gene, encoding for the tryptophan hydroxylase, has been found to exhibit a rhythmical expression in the midbrain of rats, with the highest peak arising 2 h before the light-dark transition, thus resulting in a circadian secretion of 5-HT (Malek et al., 2005). However, *Tph2* mRNA rhythm seems to be indirectly mediated by the SCN through daily glucocorticoid variation. Indeed, Malek et al. (2007) demonstrated that, in rodent MRN and DRN, the loss of corticosterone daily fluctuations, induced by adrenalectomy, abolished the rhythmical expression of *Thp2* mRNA completely. In contrast, the *Thp2* daily expression pattern was fully restored after the readjustment of plasma corticosterone levels. Nevertheless, the precise mechanisms involved in the circadian regulation of *Thp2* transcription by glucocorticoids are unknown (Malek et al., 2007). Furthermore, both serotonin transporter SERT expression and activity in mouse *raphe nuclei* have been reported to significantly change in a time-dependent manner, displaying higher levels during the dark phase (Ushijima et al., 2012). Likewise, studies on hamsters showed that 5-hydroxyindole-acetic acid (5-HIAA), the principal 5-HT metabolite, is mainly released during the dark phase (Glass et al., 1992), thus suggesting that the release of 5-HT is greater during the active phase and demonstrating that both its synthesis and release follow a circadian rhythmical pattern.

At the same time, the capability of 5-HT, not only to be controlled by the biological clock but in turn to influence circadian functions has been proven by Nakamaru-Ogiso et al. (2012) demonstrating that 5-HT deficiency in rats, induced by an acute tryptophan depletion, led to the disruption of circadian sleep-wake rhythm and disintegrated fragmental patterns. 5-HT plays an important role also in the regulation of photic entrainment of the SCN and it is able to modulate light-induced phase shifts in locomotor activity (Mistlberger et al., 2000; Cuesta et al., 2008). Indeed, 5-HT works as an inhibitor of the retinal output to the central clock, performing both a presynaptic action through 5-HT1B receptors on retinal afferent terminals, as well as a postsynaptic action through 5-HT7 receptors on SCN neurons (Smith et al., 2001). The activation of 5-HT1B receptors has also been associated with the suppression of the inhibitory effect of light on pineal melatonin production (Rea and Pickard, 2000) and with significant light-induced phase shifts, which led to a variation of SCN gene expression. The serotonergic stimulation, obtained through the treatment of the rodent model *Arvicanthis ansorgei* [a diurnal model used for the study of circadian rhythms (Hubbard et al., 2015)] with a 5-HT agonist and selective 5-HT reuptake inhibitors (SSRIs, such as fluoxetine), resulted in behavioral phase-advances and in an increase of light-induced phase-delays and advances (Cuesta et al., 2008). At the molecular level, such light-induced phase shifts, anticipated by serotoninergic activation, have been found to be correlated with changes in clock gene expression in the SCN. Notably, at CT12, *Per2*, and *Rev-erbα* mRNA levels were higher compared to those obtained after a light pulse alone, thus suggesting that they may play a role in the enhancement of light-induced phase-delays. Whereas, due to increased expression of *Per1*, *Rev-erbα*, and *Rev-erbβ* at CT0, these clock genes seemed to be implicated in the reinforcement of light-induced phase advances. On the contrary, *Rorβ* transcription was found to be reduced at CT0 (Cuesta et al., 2008).

Further evidence of the strong interconnection between circadian and serotonergic networks is related to the influence that many serotonergic drugs have on the circadian system. For example, as described before, SSRIs deeply impact the response of the circadian system to environmental light and have been seen to phase advance the firing rate of SCN neurons in rats’ brain slice cultures (Sprouse et al., 2006).

### The Noradrenergic System

Noradrenaline (NA) is a neurotransmitter mainly synthesized in the *locus coeruleus* (LC), and it is implicated in the modulation of alertness, arousal, cognition, executive functions, and mammalian circadian rhythmicity (Palm et al., 2021). The release of NA from the LC is a very dynamic process fundamental for the correct alternation between the rapid eye movement (REM) phase and the non-rapid eye movement (NREM) phase of sleep. Recent studies have revealed that the activity of LC in releasing NA is higher during wakefulness and NREM sleep, whereas it is lower during REM sleep, thus suggesting the key role of LC in sleep architecture and functions (Osorio-Forero et al., 2022). Moreover, in the LC of rats, the expression of MAO-A and MAO-B is under circadian control due to their rhythmical oscillations that peaks at nighttime, probably thanks to the presence of E-box sequences on their gene promoters (Chevillard et al., 1981).

Animal studies demonstrated that alterations of clock genes expression in the SCN, which also impact clock mRNA levels in the peripheral tissues, are associated with noradrenaline pathways. Notably, NA is able to modulate the physiological expression of *Per1*, *Per2*, and *Bmal1* in mouse livers (Terazono et al., 2003). Administration of adrenaline, both *in vitro* and *in vivo*, increased m*Per1* expression in mouse liver in a concentration-dependent manner. In SCN-lesioned arrhythmic mice, a loss in the liver daily expression of m*Per1*, m*Per2*, and m*Bmal1* and of NA content was further observed, and restored by adrenaline injections. Moreover, in human dermal fibroblasts, the exposure to NA reduced the expression of *Bmal1* at ZT12 and *Per1* at ZT20 after incubation with NA compared to controls. Meanwhile, the expression of *Per3* at CT4 was increased after the incubation with NA compared to cultures without NA treatments (Palm et al., 2021). In addition, in mice, administration of NA for 6 days highly induced *Per1* mRNA transcription in the cerebral cortex during the light phase (Burioka et al., 2008). Together, these findings demonstrate the adrenergic role in regulating clock gene expression and in conveying central clock information to peripheral organs by NA release, probably through the involvement of cAMP-PKA and MAPK-CREB pathways (Terazono et al., 2003; Burioka et al., 2008; Palm et al., 2021).

### The Glutamatergic System

Glutamate (Glu) is the primary excitatory neurotransmitter in the mammalian nervous system and it is essential for the maintenance of higher brain functions such as cognition, learning, and memory. Furthermore, it is implicated in brain cellular metabolism and in the regulation of sleep-wake states (Danbolt, 2001; Jones, 2017). Interestingly, astrocytes are primarily responsible for the rhythmic oscillation of extracellular Glu levels, which, in turn, are necessary for molecular timekeeping in the SCN. In order to regulate the balance between Glu uptake and release, astrocytes are responsible for the uptake of the majority of extracellular Glu through glutamate transporters on the surface of the astrocytic peri-synaptic processes, such as the excitatory amino acid transporters 1 and 2 (EAAT1 and EAAT2; Lehre and Danbolt, 1998). After being re-uptaken, Glu in astrocytes can be metabolized to glutamine by glutamine synthetase and used by neurons as a precursor for the synthesis of Glu or GABA (Waniewski and Martin, 1986). Otherwise, Glu can be oxidatively metabolized to α-ketoglutarate, which is involved in ATP production (Farinelli and Nicklas, 1992). On the other hand, Glu release from astrocytes is mediated by metabotropic glutamate receptors (mGluRs), which, once activated, induce an increase in the astrocytic [Ca^2+^]_i_, thus triggering Glu release from astrocytes (Hamilton and Attwell, 2010). The astroglial-released Glu activates extra-synaptic N-methyl-D-aspartate (NMDA) receptors on adjacent excitatory neurons, thus synchronizing excitatory neuronal firing (Fellin et al., 2004; Figure 2). Accordingly, the glutamatergic connections to the SCN are mediated by pre-synaptic NMDA/NR2C receptors expressed in the dorsal SCN, which, in turn, once activated by extracellular Glu, increase the GABAergic tone. Indeed, Brancaccio et al. demonstrated that inhibition of NR2C subunits of the NMDA receptors, obtained by treating mouse SCN slices with an NR2C-selective antagonist (*i.e*., DPQ-1105), led to an alteration of molecular and electrical circadian oscillations in the SCN. Notably, NR2C inhibition abolished circadian oscillations of intracellular Ca^2+^, induced nighttime depolarization of SCN dorsal neurons, reduced the amplitude, and lengthened the period of *Per2* oscillations (Brancaccio et al., 2017). Such evidence highlights that the astrocytic-Glu/NMDA-NR2C/SCN axis may represent a novel pathway required for the synchronization of SCN neurons. Moreover, in *ex vivo* studies, astrocytes of mouse SCN were found to be active and release higher Glu levels during the circadian nighttime (Brancaccio et al., 2017). Accordingly, another study demonstrated that glutamate concentration is under circadian control, and in rat brain it peaks during the dark phase of the day, mainly in the striatum and *nucleus accumbens* (Castañeda et al., 2004).

A circadian variation has also been seen in the expression of some receptors and transporters involved in the glutamate pathway. For example, Elmenhorst et al. (2016) in an *in vivo* PET experiment on rat brains, found approximately a 10% increase in the availability of metabotropic glutamate receptor mGluR5 during the light phase in different brain regions, such as the cortex, amygdala, *caudate putamen*, and *nucleus accumbens*. Meanwhile, in rodents, the glutamate transporter EAAT3, involved in Glu uptake, has been found to follow a circadian expression within the SCN, showing a peak around the dark-light transition (Cagampang et al., 1996). Brancaccio et al. (2017) demonstrated that EAAT3 inhibition, obtained by treating mouse SCN slices with a selective antagonist, led to an increase in the extracellular Glu levels, and a reduction in amplitude and robustness of *Per2* gene oscillations, resulting in an alteration of the neuronal timekeeping. Similarly, excitatory amino acid transporter 1 (EAAT1) mRNA and protein levels in the SCN, and consequently the glutamate uptake, have been found to follow a diurnal rhythm and to be strongly modulated by clock genes expression. Spanagel and coworkers found that *Per2*^Brdm1^ mutant mice showed a reduction in the SCN expression of EAAT1 compared to wild-type mice, thus resulting in a reduced glutamate uptake by astrocytes and in a hyper-glutamatergic state (Spanagel et al., 2005). Likewise, studies on cortical astrocyte cultures from *Npas2* and *Clock* mutant mice revealed a significant reduction in glutamate uptake compared to wild-type mice due to a decrease in EAAT1 mRNA levels caused by mutations (Beaulé et al., 2009). These results suggest that both Glu release and uptake are orchestrated in a circadian manner, however, the molecular mechanisms by which the clock genes modulate the total Glu uptake are unknown.

### The GABAergic System

γ-aminobutyric acid (GABA) is synthesized from Glu and it is the principal inhibitory neurotransmitter in the brain. The timing of GABA synthesis, release, and uptake seems to have a crucial role in the orchestration of SCN rhythm. Notably, the glutamic acid decarboxylase (GAD, the enzyme responsible for GABA synthesis), especially the isoform GAD65, has been reported to show a circadian oscillation. In rat SCN slices, autoradiography revealed higher levels of GAD65 mRNA in the light period rather than in the dark period, with a peak of expression during the transition from dark to light (Huhman et al., 1996). Furthermore, Maejima and coworkers demonstrated the importance of the GABAergic system for the performance of circadian behavior at the correct time of the day through the regulation of SCN neuronal activity. They found that in the SCN of Avp-Vgat^−/−^ mice (mutation resulting in a decrease of GABA release from the vesicular GABA transporter (Vgat) in arginine vasopressin-producing (AVP) neurons), the frequency of GABA-A receptor-mediated postsynaptic currents (mGPSCs) was strongly reduced during daytime compared to control mice. Besides, in the SCN of Avp-Vgat^−/−^ mice, a delay in the peak phase of *Per2:Luc* oscillations has been observed, thus leading to a lengthened activity time of circadian behavioral rhythms. Thus, these results suggest the key role of the GABAergic transmission from AVP neurons in directing the correct timekeeping of SCN outputs from molecular clocks to behavior (Maejima et al., 2021).

Further evidence for GABA involvement in the induction of a rhythmic expression of the core clock genes in neurons, through the activation of GABA-A receptors, comes from Barca-Mayo et al. (2017). In *in vitro* cortical neurons of GABA-treated mice, they found that GABA was able to entrain rhythmic oscillations of *Bmal1*, *Cry1*, and *Per2* expression, and that the inhibition of GABA-A receptor signaling suppressed such rhythms. In *Bmal1cKO* mice (inducible knockout mouse model of *Bmal1* in astrocytes), extracellular GABA levels have been found to be altered, resulting in an over-inhibition of the signaling pathways involved in cognitive functions and in a delay of the active phase. The impairment of GABA levels has been related to the deletion of astrocytic *Bmal1* that decreased the expression of GABA transporters *Gat1* and *Gat3* in the SCN and in astrocytes, in turn reducing extracellular GABA clearance (Barca-Mayo et al., 2017). Furthermore, also the glial deletion of *Per2* gene in mice (G*Per2* mouse model) has been found to alter *Gats* mRNA levels (Martini et al., 2021). Notably, the expression of *Gat1* significantly decreased in the hypothalamus of G*Per2* mice, whereas that of *Gat2* has been reported to increase in the NAc of this mouse model compared to controls (Martini et al., 2021). Together, these findings suggest that astrocytic *Bmal1* and glial *Per2* may regulate the extracellular GABA concentration through the modulation of *Gats* mRNA levels.

Moreover, GABA is also important in the regulation of retinal clock genes fluctuations, since, in cultured m*Per2*^Luc^ retinal explants, it decreased and suppressed the rhythmic amplitude of *Per2* oscillations in a dose-dependent manner, possibly through the activation of GABA-A and GABA-C receptors (Ruan et al., 2008). Only after treating m*Per2*^Luc^ retinal explants with a GABA-A or GABA-C receptor agonist, the amplitude of *Per2:Luc* signals significantly decreased, whereas no effects have been observed with GABA-B receptor agonists (Ruan et al., 2008). Moreover, GABA was able to increase *Per1* and *Bmal1*, but not *Per2* and *Clock* mRNA levels, thus indicating its ability to suppress *Per2* levels acting at the posttranscriptional level, by stimulating casein kinase, which is able to phosphorylate *Per2* for its ubiquitin-mediated proteasomal degradation (Ruan et al., 2008).

## The Role of Glia in The Generation of Circadian Rhythmicity

The existence of an endogenous neuronal circadian timekeeper with the capacity to orchestrate and coordinate physiological and behavioral processes with the external environment is now well established. However, studies on circadian timekeeping are moving beyond the neuronal cell population, since the capability of encoding rhythmical information is not only restricted to neurons but also extends to glial cells. Indeed, as discussed above, the devolution of the control of the glutamatergic and, consequently, of the GABAergic tone to astrocytes, since they are the primarily responsible for the rhythmic oscillation of extracellular Glu levels, suggests that such glial cells synergistically communicate with neurons to establish the 24 h daily rhythm (Figure 2). Hence, since astrocytes and microglia are integral parts of the timepiece, the correct functioning of the glial clock system is fundamental for the maintenance of a healthy brain. Recent studies indicate that misalignments in the expression of glial clock genes contribute to the development of different pathological outcomes. Particularly, astrocytic *Clock*, *Per1*, and *Per2* have been found to be involved in the modulation of ATP release (Marpegan et al., 2011) and, subsequently, in the regulation of energy metabolism and glial activity. Therefore, mutations of these clock genes may result in metabolic diseases. Moreover, microglial *Bmal1* has been proven to be an important molecular player in the inflammatory response and in different pathological processes, such as neurological diseases and sleep disorders. Notably, mouse microglial *Bmal1* deficiency has been found to decrease the expression of pro-inflammatory cytokines (*i.e*., IL-1β, IL-6, and TNFα) and increase that of anti-inflammatory cytokines (*i.e*., IL-10; Wang et al., 2020), probably owing to the presence of E-boxes sequences on cytokine gene promoters. Likewise, the deletion of mouse astrocytic *Bmal1* leads to the impairment of cognitive functions, majorly affecting the declarative memory (Barca-Mayo et al., 2017), as well as to an imbalance of the GABAergic system (Barca-Mayo et al., 2017), resulting in the development of neurological and sleep disorders. In this regard, another core glial clock component was revealed to play an important role in the regulation of mood-related behaviors, such as anxiety and depression (Martini et al., 2021). G*Per2* mice, in fact, showed significantly less immobility in the forced swim test, thus displaying reduced despair, one of the main manifestations of depression (Martini et al., 2021). Besides, they spent more time in the open field (an anxiety-provoking space) of the elevated O-maze, indicating a reduced anxiety-like behavior in mice lacking *Per2* in glial cells (Martini et al., 2021). Nevertheless, glial *Per2* KO had no influence on circadian parameters since G*Per2* mice did not display any differences in the wheel-running activity and in body temperature fluctuations compared to control animals, thus indicating that the deletion of *Per2* in glial cells did not affect free-running rhythms in mice (Martini et al., 2021). An interesting observation is that, contrary to glial *Per2* KO animals, total *Per2* KO mice exhibited a shortening of the free running period (Martini et al., 2021). Therefore, these data suggest the importance of glial *Per2* in the regulation of mood behaviors through a process which, given the normal maintenance of circadian parameters, is not associated with the circadian clock machinery, but is probably related to the action of glial *Per2* on the glutamatergic, GABAergic, and dopaminergic signaling pathways (Martini et al., 2021).

Collectively, these findings suggest that an abnormal circadian system in glial cells may degenerate into metabolic diseases, immune system dysfunctions, and psychiatric illnesses. Therefore, deepening the knowledge of the role of the glial population in the generation of rhythm may be important for the discovery of new pathways and interactors involved in the development of these pathological disorders. In the following section, we will summarize recent findings reporting the existence of an autonomous rhythmical pattern in glia cells, demonstrating that astrocytes and microglia are not passive participants in the orchestration of rhythm, but play a key role in the regulation of circadian rhythms and in the maintenance of timekeeping within the brain (Hayashi et al., 2013; Fonken et al., 2015; Barca-Mayo et al., 2017; Brancaccio et al., 2017, 2019; Nakazato et al., 2017; Sominsky et al., 2021).

### Astrocytes-Neurons Interaction in the Circadian Rhythmicity

Considerable evidence supports the primary involvement of astrocytes in the generation of rhythm, affirming their capacity to influence the molecular clock machinery of the central circadian pacemaker (Barca-Mayo et al., 2017; Brancaccio et al., 2017, 2019). Astrocytes express clock genes and can act as autonomous circadian oscillators (Ewer et al., 1992; Prolo et al., 2005). Ewer et al. (1992) first demonstrated the existence of a circadian clock in glia, by reporting the presence of the core clock component *period* in the astrocytes in *Drosophila melanogaster*. Also, primary cultures of cortical astrocytes derived from *Per2::luciferase* knock-in mice and *Per1::luciferase* transgenic rats were found to express circadian rhythms in the activity of these two clock genes, with a longer average period in the activity of *Per1::luc* than *Per2::luc* (Prolo et al., 2005). Furthermore, beyond Glu, also some gliotransmitters, such as adenosine triphosphate (ATP), a neuronal transmitter involved in synaptic communication between astrocytes and neurons, are released following a circadian pattern (Womac et al., 2009; Marpegan et al., 2011). In particular, similarly to that observed in SCN cell cultures, extracellular ATP release and accumulation followed a daily rhythm in primary cultures of rat cortical astrocytes, thus suggesting that ATP might represent an important output of the mammalian molecular clock (Womac et al., 2009). Indeed, extracellular ATP levels have been found to strictly depend on clock genes expression. In this context, mouse astrocytes carrying mutations on *Clock*, *Per1*, and *Per2* genes showed a constitutively blunted ATP fluctuation during the day compared to wild-type cultures (Marpegan et al., 2011). Moreover, astrocytes have been suggested to be active during the circadian night, in contrast to neurons, whose peak of activity has been registered during the circadian day. These findings are based on data reporting that, in mouse SCN astrocytes, Glu release peaked during the nighttime in phase with astrocyte [Ca^2+^]_i_ levels, that were anti-phasic to those of neuronal [Ca^2+^]_i_ (Brancaccio et al., 2017). Overall, these results underline the ability of the astrocyte clock to impose its own rhythm autonomously, thus indicating that astrocytes are actively involved in the maintenance and control of molecular timekeeping within the brain.

However, such rhythmicity of astroglia is partly entrained and sustained by the SCN master clock (Prolo et al., 2005). In co-cultures of mouse cortical astrocytes with adult SCN explants, the amplitude of *Per1* rhythmicity has been observed to be significantly greater than that of primary astrocytes alone (Prolo et al., 2005), thus suggesting the existence of a crosstalk between the master neuronal clock in the SCN and the glial clock. In particular, in addition to SCN neurons, also astrocytes are able to encode circadian information and have been observed to strongly influence the central circadian pathway and the master clock machinery (Brancaccio et al., 2017, 2019; Tso et al., 2017). Notably, the increased release of Glu by astrocytes during the night has been found to be accomplished by a decrease in *Per2* expression rhythms in the SCN, thus denoting the active role played by astrocytes in promoting correct molecular timekeeping in the SCN *via* the glutamatergic signaling (Brancaccio et al., 2017). Furthermore, Brancaccio et al. (2019) using SCN slices of *Cry1/2*-null mice, demonstrated that the expression of *Cry1* alone in astrocytes was sufficient to initiate self-sustained circadian rhythms of *Per2* in the SCN, thus restoring the central molecular clock that was previously repressed, though with a longer time extent than neuronal *Cry1*. The crosstalk between SCN and astrocytes has also been suggested by data on astrocytes affecting the circadian pattern of behavior in mice. Although astrocytes are not directly connected to motor centers, the rhythmicity of locomotor activity, completely lost in *Cry1/2*-null mice, was re-established after the *Cry1* transfection of SCN astrocytes (Brancaccio et al., 2019), thus speculating that the astrocytic TTFL indirectly drives new circadian behaviors by hiring and restoring the neuronal TTFL in the SCN, probably by recruiting the E-box-based TTFL of neurons through the driving of neuronal [Ca^2+^]_i_ oscillations (Brancaccio et al., 2019).

In addition, *Bmal1* has also been reported to follow a rhythmical pattern and to strictly impact the circadian communications between astrocytes and neurons (Barca-Mayo et al., 2017; Tso et al., 2017). *Bmal1* deletion in astrocytes has been found to lead to a lengthening of *Per2* and locomotor circadian periodicity (Tso et al., 2017). Moreover, when maintained in constant darkness, a delay and reduction in the active period have been observed in *Bmal1* KO mice, as well as an impairment in cognitive functions mainly affecting the declarative memory (Barca-Mayo et al., 2017). Indeed, *Bmal1* KO mice were subjected to the novel object recognition (NOR) test, where they were exposed to two identical objects for 10 min, and they were subsequently tested by changing one of the familiar objects with a new one after 1 h and 24 h, with the aim to assess short-term and long-term memory respectively (Barca-Mayo et al., 2017). As a result, *Bmal1* KO animals displayed a significant reduction in the discrimination index after both 1 and 24 h, thus demonstrating both short-term and long-term memory being compromised (Barca-Mayo et al., 2017). Notably, oscillations of *Cry1*, *Bmal1*, and *Per2* expression were significantly impaired in the cortex and hippocampus of astrocyte *Bmal1* KO mice, thus demonstrating the existence of a strong intercellular communication and interplay between astrocytes and neurons that influence the circadian time-keeping within the brain and is fundamental for the maintenance of a healthy brain (Barca-Mayo et al., 2017).

Further demonstration of the ability of astrocytes to encode circadian information, and thus to influence the neuronal clock machinery, was provided by the finding that, in co-cultures of mouse synchronous astrocytes with asynchronous primary cortical neurons, the former was able to re-establish the rhythmical expression of *Bmal1*, *Cry1*, and *Per2* in the arrhythmic cortical neurons (Barca-Mayo et al., 2017). In contrast, astrocytes with *Bmal1* deletion failed to induce rhythmicity in the clock genes expression of asynchronous cortical neurons, thus demonstrating the pivotal role of an active astrocyte clock for adequately affecting the neuronal clockwork (Barca-Mayo et al., 2017). Accordingly, the deletion of astrocytic *Bmal1* has been found to impact the neuronal clock through GABA signaling, which acts as a mediator between neuron and astrocyte communication. In co-cultures of astrocytes with asynchronous cortical neurons, the inhibition of the GABA-A receptors prevented the astrocytic induction of a sustained rhythm in neurons, suggesting that the GABAergic pathway is required for the transmission of rhythm from astroglia to neurons (Barca-Mayo et al., 2017). Indeed, in astrocytes with *Bmal1* KO, the expression of GABA transporters *Gat1* and *Gat3* significantly decreased in the cortex and in the SCN compared to WT astrocytes, thus impairing the extracellular levels of GABA, providing the explanation for the reason behind the failure of the arrhythmic astrocytes to synchronize the clock of asynchronous cortical neurons (Barca-Mayo et al., 2017). All these data suggest that the loss of daily rhythm in astrocytes due to the deletion of astrocytic *Bmal1* is sufficient to lengthen and impair circadian rhythms in neurons and in behavior, both *in vitro* and *in vivo*, demonstrating that astrocytes may actively contribute to the generation of SCN neuronal output rhythm and to internal clock synchronization.

### Microglia: The Rhythmicity Around Immune and Inflammatory Response

Circadian clock genes are rhythmically expressed in microglia in several brain areas, such as the hippocampus and the cortex (Hayashi et al., 2013; Fonken et al., 2015; Nakazato et al., 2017). In hippocampal microglia isolated from adult rats, *Per1* and *Per2* expression was found to reach its peak in the middle of the light phase, *Rev-erb* at the end of the light phase, whereas the higher level of *Bmal1* expression was observed at the onset of the light period and in the middle of the dark phase (Fonken et al., 2015). Furthermore, also murine cortical microglia showed a fluctuant expression of *Per1, Per2*, *Rev-erbα*, and *Npas2*, thus supporting the idea of the existence of an autonomous clock machinery in microglia (Nakazato et al., 2011; Hayashi et al., 2013; Nakazato et al., 2017). The presence of the core clock proteins in microglia may also be responsible for the rhythmicity of a variety of microglial functions, such as immune and inflammatory response, by tuning the rhythmic fluctuations in basal inflammatory genes expression, such as cytokines and chemokines (Saijo and Glass, 2011). In rat hippocampal microglia, the expression of interleukin-1β (IL-1β), interleukin-6 (IL6), interleukin 1R1 (IL1R1), and tumor necrosis factor α (TNFα) was strictly affected by the time of the day, and, similarly to clock genes, they were strongly expressed during the light phase (Fonken et al., 2015). In addition, *Bmal1* has been found to be a critical player in the immunological activity of microglial cells, possibly due to the presence of E-box sequences in gene promoters of several cytokines (Nakazato et al., 2017; Wang et al., 2020). In particular, microglial *Bmal1* was found to be a key mediator for the regulation of the expression of IL6 (Nakazato et al., 2017). Accordingly, *Bmal1* deficiency has been found to decrease the expression of pro-inflammatory cytokines (*i.e*., IL-1β, IL-6, and TNFα) and increase that of anti-inflammatory cytokines (*i.e*., IL-10) in mouse microglia (Wang et al., 2020). These data support the involvement of the microglial clock system in the regulation of inflammatory responses. Another microglia-associated function with diurnal rhythmicity is the regulation of synaptic strength *via* the circadian secretion of cathepsin S (CatS). CatS is a microglia-specific lysosomal cysteine protease rhythmically secreted during the dark phase, fundamental for the maintenance of neuronal circuitry (Hayashi et al., 2013). Hayashi and coworkers found that CatS expression followed a circadian pattern driven by the intrinsic microglial molecular clock (Hayashi et al., 2013). Accordingly, in WT mouse cortical microglia, CatS transcripts showed a peak at CT14, whereas, in the cortical microglia of *ClockΔ19* mutant mice, no diurnal fluctuation of CatS expression was observed, thus suggesting that its transcription was activated and mediated by the binding of *Clock:Bmal1* heterodimer to the E-boxes sequences on the CatS gene promoter (Hayashi et al., 2013). All these data demonstrate the existence of a microglial clock involved in the regulation of microglia-mediated functions, including the neuroimmune roles and the control of the synaptic strength.

Beyond increasing knowledge regarding the presence of an intrinsic clock in microglial cells, data from literature report that, in turn, the microglial clock system may also influence the neuronal clock machinery and rhythms among the CNS. Indeed, ablation of microglia resulted in the disruption of the central circadian system both at molecular and physiological levels (Sominsky et al., 2021). Notably, using Cx3cr1-Dtr rats, a rat model in which microglia is depleted, aberrations in clock gene expression were observed (Sominsky et al., 2021). In the SCN, *Per1* mRNA levels were increased and a reduction in *Bmal1* protein was revealed, whereas, in the hippocampus, the microglial depletion led to a suppression of *Bmal1* expression and to an increase of *Per1* and *Per2* mRNA levels (Sominsky et al., 2021). Moreover, ablation of microglial cells from the CNS led to a disruption of body temperature rhythms, energy metabolism, and diurnal activity profiles. To such effect, Cx3cr1-Dtr rats showed a significant reduction in total energy expenditure during their active phase and also in their diurnal rhythms compared to WT (Sominsky et al., 2021). Accordingly, Sominsky et al. (2021) also observed a total loss of diurnal activity rhythms and a changing in temperature rhythms, with a pattern completely inverse to that of energy expenditure and activity changes. Considered together, these findings suggest that microglia may play a key role in diurnal rhythm generation since its ablation leads to the concomitant disruption of diurnal profiles and to the dysregulation of the expression of certain clock genes in different brain domains. In contrast, Barahona and collaborators showed that, in the cortex, ablation of microglia did not affect the rhythmical pattern of clock genes expression, since in Cx3cr1-Dtr rats clock genes all maintained their diurnal rhythmical expression (Barahona et al., 2022). Due to these conflicting data, further studies on this glia population are required to elucidate its role in the generation of rhythms throughout the brain.

## A Matter of Time: Why Understanding The Functioning of The Biological Clock in The Cns Is Crucial?

**Figure 3:**
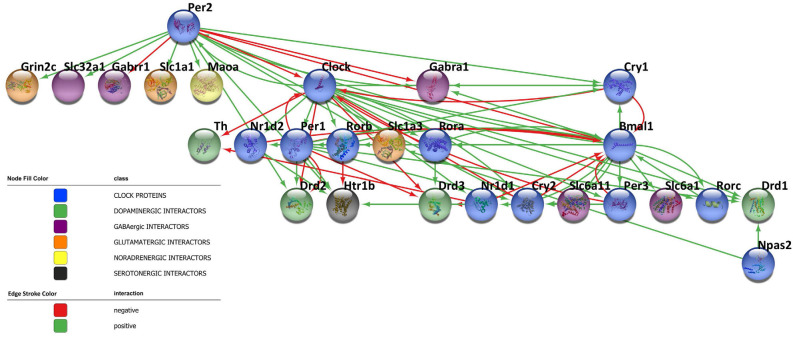
Bidirectional crosstalk between clock proteins and neurotransmitter players. The network contains 27 nodes and the edges represent the crosstalk between the clock proteins and neurotransmitter players depicted by the literature (positive regulation: green line, negative regulation: red line). The different biological processes investigated are shown in the network according to the color legend. The network has been visualized using Cytoscape 3.9.1 (Shannon et al., 2003).

Circadian rhythms are dictated by highly specialized cells of specific brain areas that orchestrate a complex network of coupled self-sustained clocks both in the brain and in peripheral organs. Remarkably, neurons are not the only CNS cell population to orchestrate the rhythm, but also astrocytes and microglia have been reported to harbor an intrinsic autonomous clock and to actively participate in the maintenance of timekeeping within the brain. Moreover, since neurotransmitters play a key role in the diffusion of circadian information among CNS cells and impact the core clock machinery, they have emerged as fine-tuners of daily rhythmicity. Indeed, the bidirectional interplay between neurotransmitters and the circadian clockwork is fundamental to maintain the accuracy and precision in daily timekeeping among different brain areas. In this regard, the network reported in Figure 3 shows the complex interactions taking place between clock proteins and several neurotransmitter players, as depicted by the literature discussed above, with the aim of illustrating this intricate bidirectional crosstalk (Figure 3). However, much is still unknown about the deeper molecular interconnections involved in the orchestration of the temporal variable among CNS cells. Therefore, expanding our knowledge about this complex network appears fundamental to better understand the functioning of the biological clockwork, which represents an extremely dynamic process across the lifespan. Indeed, rhythmic activities have been observed to significantly change during the pace of aging, also contributing to the acceleration of the process. Furthermore, due to the sexually dimorphic nature of some neurotransmitters systems [*e.g*., the NA system (Joshi and Chandler, 2020), the DA system (Rial et al., 2018), and the 5HT system (Näslund et al., 2013)], the connection between chemical transmission and expression and functions of clock genes in the CNS might account for sex-dependent variability in the circadian timing system. In this regard, seasonality, as well as most of the sex and gender-related-mechanisms, is known to affect the efficiency of learning, memory, and mood control processes (Bailey and Silver, 2014; Urban et al., 2021). Although so far poorly investigated, the interplay linking sex-neurotransmitters-clock genes deserves of course attention, since if disrupted or malfunctioning, might subserve the development of central disorders including those that so far preferentially attracted the interest of the researchers as discussed below.

Noteworthy, although future longitudinal studies are necessary to corroborate such preliminary evidence, the circadian system seems to play a key role in the pathogenesis of psychiatric diseases and neurodegenerative disorders. In particular, circadian rhythms disruption has been described not only as an early warning sign of neurodegenerative diseases but also as a potential risk factor. Evidence from literature suggests the existence of a strict correlation between several neurodegenerative diseases, specifically Alzheimer’s disease (AD) and Parkinson’s disease (PD), and circadian rhythms destruction (CRD; Videnovic et al., 2014). Accordingly, AD and PD share common features and symptoms ascribable to an abnormal and disrupted circadian pattern (Videnovic et al., 2014). For example, patients with neurodegenerative diseases show frequent disorders in the sleep/wake cycle, with sleep fragmentation and an increase in activity levels in the nighttime, and a prevalence of sleepiness during daytime (Merlino et al., 2010; Breen et al., 2014). Notably, it has been confirmed that an alteration of the molecular clockwork leads to an increase in the oxidative injury of the brain, thus promoting neuronal damage, cell death, and neurodegeneration. Interestingly, several studies on mice found that both global and brain-specific deletion of *Bmal1* induced higher neuronal oxidative damage, synaptic degeneration, severe astrogliosis, impaired neuronal network functional connectivity, and impaired learning and memory, compared to age-matched wild type controls, resulting in premature aging, reduced lifespan, and pathogenesis of neurodegeneration (Musiek et al., 2013). Furthermore, the majority of neurodegenerative disorders are associated with the accumulation of intra- and extra-cellular aggregates (*i.e*., amyloid-β, phosphorylated tau protein, α-sinuclein), whose clearance is partly controlled by the glymphatic flow. Recent studies on mouse models have found that the glymphatic flow follows a rhythmical circadian pattern with an important upregulation during sleep, thus facilitating the removal of metabolic products accumulated during wakefulness (Hablitz et al., 2020). Remarkably, in mouse models of neurodegenerative disorders (such as APP/PS1 mice, a model of AD), where circadian rhythms are disturbed, a reduced brain clearance has been reported, due to the presence of disrupted flow patterns of the glymphatic system, thus resulting in a continuous and augmented accumulation of aggregates that, in turn, may promote cell death and degeneration of brain structures (Peng et al., 2016; Rasmussen et al., 2018). In conclusion, expanding our knowledge about the molecular regulation of circadian rhythmicity in CNS cells within different brain areas represents a crucial challenge, with a potential impact on the biological comprehension of the onset of neurodegenerative processes.

## Author Contributions

FF and CL: conceived the idea. FF, EB, AP, and CL: wrote the manuscript. EP: network analysis. FF, EB, AP, EP, CS, SG, and CL: critical discussion. All authors contributed to the article and approved the submitted version.

## Conflict of Interest

The authors declare that the research was conducted in the absence of any commercial or financial relationships that could be construed as a potential conflict of interest.

## Publisher’s Note

All claims expressed in this article are solely those of the authors and do not necessarily represent those of their affiliated organizations, or those of the publisher, the editors and the reviewers. Any product that may be evaluated in this article, or claim that may be made by its manufacturer, is not guaranteed or endorsed by the publisher.
